# Prognostic value of pretreatment albumin/globulin ratio in digestive system cancers: A meta-analysis

**DOI:** 10.1371/journal.pone.0189839

**Published:** 2018-01-04

**Authors:** Hui-Wen Guo, Tang-Zhan Yuan, Jia-Xi Chen, Yang Zheng

**Affiliations:** Department of General Surgery, The Fourth Affiliated Hospital of Nan Chang University, Nanchang, Jiangxi, China; University Hospital Llandough, UNITED KINGDOM

## Abstract

The albumin/globulin ratio (AGR) has been widely reported to be a potential predictor of prognosis in digestive system cancers (DSCs), but convincing conclusions have not been made. Therefore, herein, we performed a meta-analysis of relevant studies regarding this topic to evaluate the prognostic value of AGR in patients with DSCs. Three databases, including PubMed, EMBase, and Web of science, were searched comprehensively for eligible studies through September 8, 2017. The outcomes of interest included overall survival (OS), disease-free survival (DFS), and cancer-specific survival (CSS). In our meta-analysis, pooled analysis of 13 studies with 9269 patients showed that a low AGR was significantly correlated with poor OS (HR = 1.94; 95% CI: 1.57–2.38; P <0.001). Five studies with 6538 participants involved DFS, and our pooled analysis of these studies also demonstrated that there was a significant association of a low AGR with worse DFS (HR = 1.49; 95% CI: 1.10 to 2.00; P < 0.001). In addition, only 2 studies referred to CSS, and we also detected a significant relationship between a low AGR and worse CSS from the results of our meta-analysis. In summary, a low pretreatment AGR was related to unfavorable survival in human digestive system cancers. A low pretreatment AGR may be a useful predictive prognostic biomarker in human digestive system cancers.

## Introduction

Digestive system cancers are threaten human life and health [1]. A recent study reported that the incidence rates of several digestive system cancers, including hepatocarcinoma, esophagus carcinoma, pancreatic cancer and intestinal malignancies, have a tendency to be elevated, although cancer-associated death has been continuously decreasing over the past two decades[2]. Thus, the epidemiology of digestive system cancers (DSCs) remains grim. Several poor clinicopathological characteristics, such as advanced clinical stage, poor tumor differentiation and larger tumor size, were popularly recognized to be significantly associated with an unfavorable prognosis, but patients with similar clinicopathological characteristics often suffer different survival outcomes[1]. Hence, identification of new reliable biomarkers that can more precisely predict the prognosis of patients with DSCs is very imperative.

It has been reported that abnormal serum albumin is closely related to the progression of many diseases [3]. Furthermore, previous studies have demonstrated that a decreased albumin level is correlated with an unfavorable prognosis of DSCs, including gastric, colorectal, and pancreatic cancers [4–7]. In addition, serum globulin has also been demonstrated to have a close relationship with immunity and inflammation and acts as a valuable predictor of tumor progression[8]. However, both serum albumin and globulin levels are easily influenced by non-cancer-related factors, including dehydration and edema, which can weaken their efficiency and accuracy in predicting the prognosis of cancer patients. To overcome this defect, numerous recent studies have tried to investigate the prognostic significance of the albumin/globulin ratio (AGR) in cancer patients, which combines two factors and thus might reduce the influence of confounding factors. For instance, a body of studies reported that a low pretreatment AGR is closely associated with worse prognosis in patients with digestive system cancers, such as gastric cancer[9, 10], colorectal cancer [11–15], pancreatic cancer[16], hepatocellular carcinoma[17] and so on. However, most studies published to date use a small sample size, which might affect the reliability of their conclusions. Therefore, it is imperative to perform a meta-analysis of studies investigating the prognostic value of the AGR in patients with DSCs to provide stronger evidence in favor of the prognostic value of the AGR.

## Methods

### Literature search strategy

A systematic literature search was performed in PubMed, EMBase and Web of science for eligible studies assessing the prognostic value of the AGR in digestive system cancers through September 8, 2017. The search strategy combined the following terms: (gastric or stomach or colon or rectal or colorectal or liver or hepatocellular or pancreatic or esophageal or esophagus or cholangio* or gallbladder or bile duct) and (tumor or cancer or carcinoma or adenocarcinoma or malignan*) and (“albumin to globulin ratio” or “albumin/globulin” or “albumin to globulin” or “AGR” or “albumin and globulin”), as well as (prognosis or prognostic or survival).

### Selection criteria

The inclusion criteria for eligible studies included: (1) reported the association between the AGR and OS/DFS/CSS in digestive system cancers; (2) had full text and were published in English; and (5) had a hazard ratio (HR) with a 95% confidence interval (CI) or a survival curve. Furthermore, studies were excluded according to the following criteria: (1) case reports, reviews, letters and comments; (2) no sufficient data could be extracted to calculate the HR and 95% CI; (3) patients were not divided into two groups, including a low AGR group and high AGR group; and (4) studies were performed on animals.

### Data extraction and quality assessment

The full texts of the included studies were carefully reviewed, and data were extracted by two independent researchers. A third investigator consulted to resolve inconsistencies. The following data were extracted: the first author’s name, country of research, year of publication, mean age of patients, cancer type, case number, study type, cut-off AGR, cut-off selection, treatment method, mean follow-up time, overall survival (OS), cancer specific survival (CSS), and diseases free survival (DFS). If the included studies provided both univariate and multivariate analysis results, only the multivariate results were extracted since they balanced many confounding factors. When HRs for OS, CSS and DFS could not be obtained directly, Engauge Digitizer version 4.1 (http://digitizer.sourceforge.net/, freely down-loaded software) was used to extract them from the Kaplan-Meier curves.

The Newcastle-Ottawa Scale (NOS) was used to assess the study quality. Three items were evaluated, selection, comparability and outcome [18]. The scores according to NOS varied from 0 to 9. A score of 6 or more was recognized as high quality.

### Statistical analysis

Statistical analyses of this meta-analysis were fulfilled using Stata version 12.0 (Stata Corporation, College Station, TX, USA). Pooled HRs and 95% CIs were used to evaluate the quantitative aggregation of the survival results. The heterogeneity across studies was tested by Cochran’s Q and Higgins I^2^ statistics. P < 0.05 and I^2^>50% were considered to be significant heterogeneity, while I^2^ < 25% and 25% < I^2^ < 50% indicated no heterogeneity and moderate heterogeneity, respectively. A random effects model was used when statistical heterogeneity was detected (P < 0.05, I^2^ > 50%); otherwise, the fixed effects model was applied. HR > 1 (high AGR used as reference) means a higher risk of worse outcomes for low AGR, and the study was recognized as statistically significant if the 95% CI did not include 1 and P<0.05. Sensitivity analysis was carried out by sequentially omitting individual studies at each step. If the results did not substantially alter when one study was excluded, this meant that the pooled results were stable. Publication bias was evaluated by Begg’s test, Egger’s test and funnel plot analysis, and P < 0.05 with funnel plot asymmetry indicated that a statistically significant publication bias may exist [19, 20]. Duval’s nonparametric trim-and-fill method was used to evaluate the potential effect of publication bias [21], if significant publication bias exist.

## Results

### Study search and study characteristics

**Fig 1:**
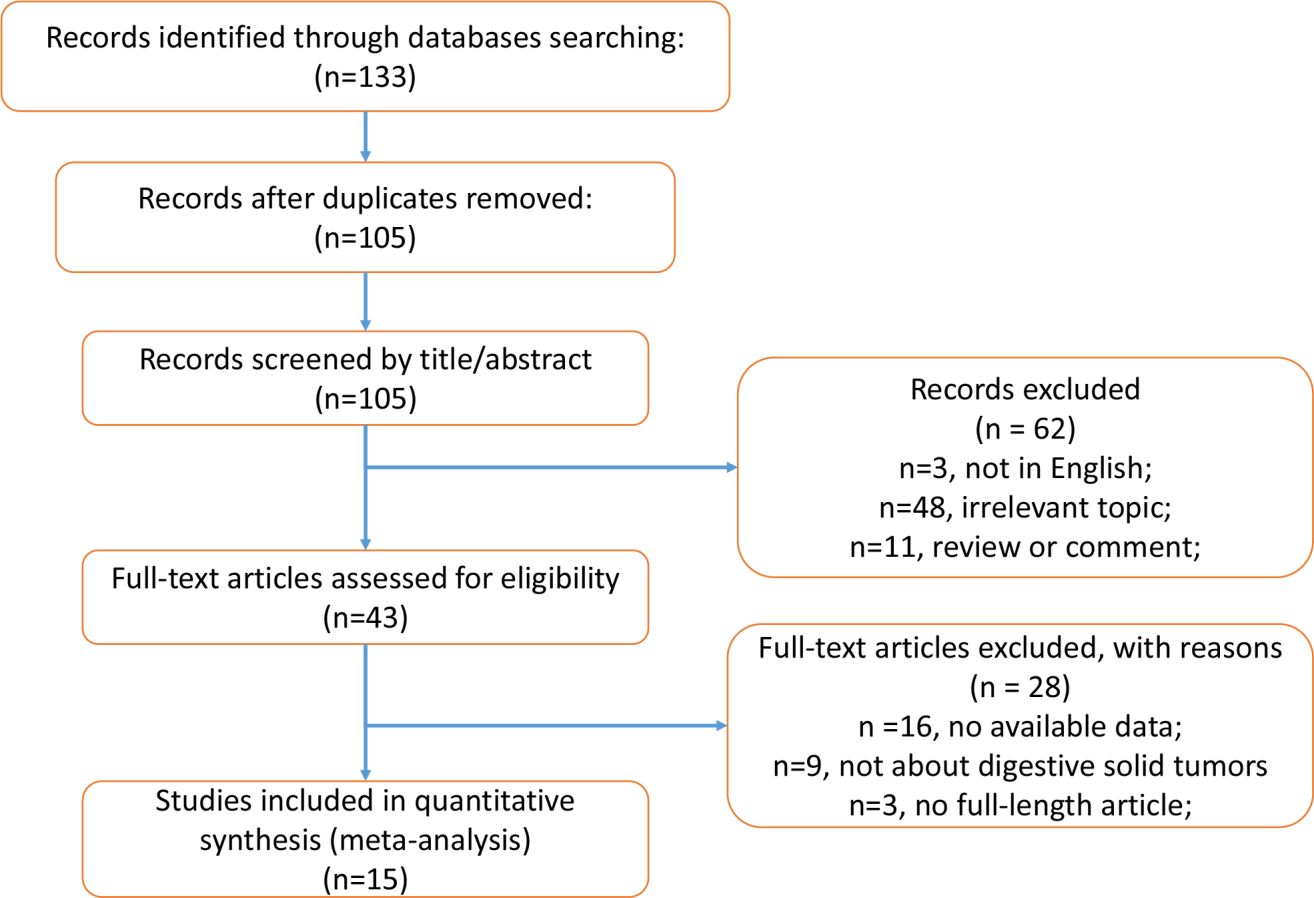
Flow diagram of study selection.

The search and selection strategy is shown in Fig 1. A total of 133 studies were identified after searching PubMed, EMBase and Web of science. After 28 duplicates were removed, 105 studies were checked by two investigators who screened the titles and abstracts. Subsequently, 62 studies were excluded because they were reviews and comments (n = 11), covered irrelevant topics (n = 48) and were not published in English (n = 3), and 43 studies were left for full-text review. Finally, 15 studies were included in this meta-analysis after 16 excluding publications with no available data, 9 studies regarding non-digestive malignancies and 3 articles without full-length text. The sample sizes of these studies ranged from 66 to 5336. Among these studies, 5 studies were on colorectal cancer [11–15], 4 studies involved gastric cancer [9, 10, 22, 23], 3 studies referred to esophageal cancer[8, 24, 25], and the rest of the included studies were on pancreatic cancer[16], liver cancer[17] and cholangiocarcinoma [26]. With respect to the study region, 10 studies were performed in China, 4 in Japan, and only one in the USA. The study period of the 15 studies ranged from 2013 to 2017. All of the included studies were designed retrospectively. According to the Newcastle–Ottawa scale (NOS), the study quality score varied from 5 to 7, which indicated that the study quality was moderate to high. The information above and other characteristics of the included studies are presented in Table 1.

**Table 1:** Characteristics of included studies.

Study	Country	Year	Age (years)	Cancer type	Initial metastasis	Case number	Study type	Cut-off of AGR		Cut-off Selection	Treatment methods	Follow- up (Median months)	NOS score
								Low level	High level				
Azab	USA	2013	68.85	Colorectal cancer	N	356	R	<1.028	>1.321	Tertiles	Surgery, Chemotherapy	40	6
Jie Chen	China	2015	61	Gastric cancer	N	186	R	≤1.33	>1.33	X-tile program	Surgery	NR	5
FUJIKAWA	Japan	2017	70	Colon cancer	N	248	R	<1.32	≥1.32	ROC	Surgery, Chemotherapy	49.7	7
Qingguo Li	China	2015	54	Rectal cancer	N	293	R	≤1.20	>1.20	X-tile program	Radiotherapy, Chemotherapy, Surgery	NR	6
Xiao-Hui Li	China	2017	59	Esophageal Carcinoma	N	447	R	<1.66	≥1.66	ROC	Surgery	52.0	6
Yaqi Li	China	2016	59	Colorectal cancer	N	5,336	R	<1.50	≥1.50	NR	Surgery, Chemotherapy	55.2	7
Jianjun Liu	China	2017	58.8	Gastric cancer	N	507	R	<1.93	≥1.93	X-tile program	Surgery	NR	6
Lin	China	2017	58	Cholangiocarcinoma	N	123	R	≤1.44	>1.44	ROC	Surgery	14.8	5
Mao	China	2017	59	Gastric cancer	N	862	R	≤1.50	>1.50	R language	Surgery	NR	5
Oki	Japan	2017	68	Esophageal cancer	N	112	R	≤1.41	>1.41	ROC	Surgery, Chemotherapy, Radiotherapy	24.9	6
Shibutani	Japan	2015	63	Colorectal cancer	Y	66	R	>1.25	≤1.25	ROC	Chemotherapy	NR	5
Toiyama	Japan	2017	67	Gastric cancer	N	384	R	≤1.3793	>1.3793	ROC	Surgery, Chemotherapy	47.6	7
Jie Xu	China	2016	58.4	Pancreatic adenocarcinoma	N	265	R	≤0.9	>0.9	X-tile program	Surgery	NR	6
Jiahe Zhang	China	2016	49	Hepatocellular carcinoma	N	105	R	<1.18	≥1.18	Cutoff Finder	Surgery	NR	5
Fei Zhang	China	2016	59	Esophageal carcinoma	N	458	R	<1.30	≥1.30	Cutoff Finder	Surgery, Chemotherapy, Radiotherapy	NR	6

### Prognostic value of the AGR in digestive system cancers

#### A low AGR and overall survival (OS) in digestive system cancers

**Fig 2:**
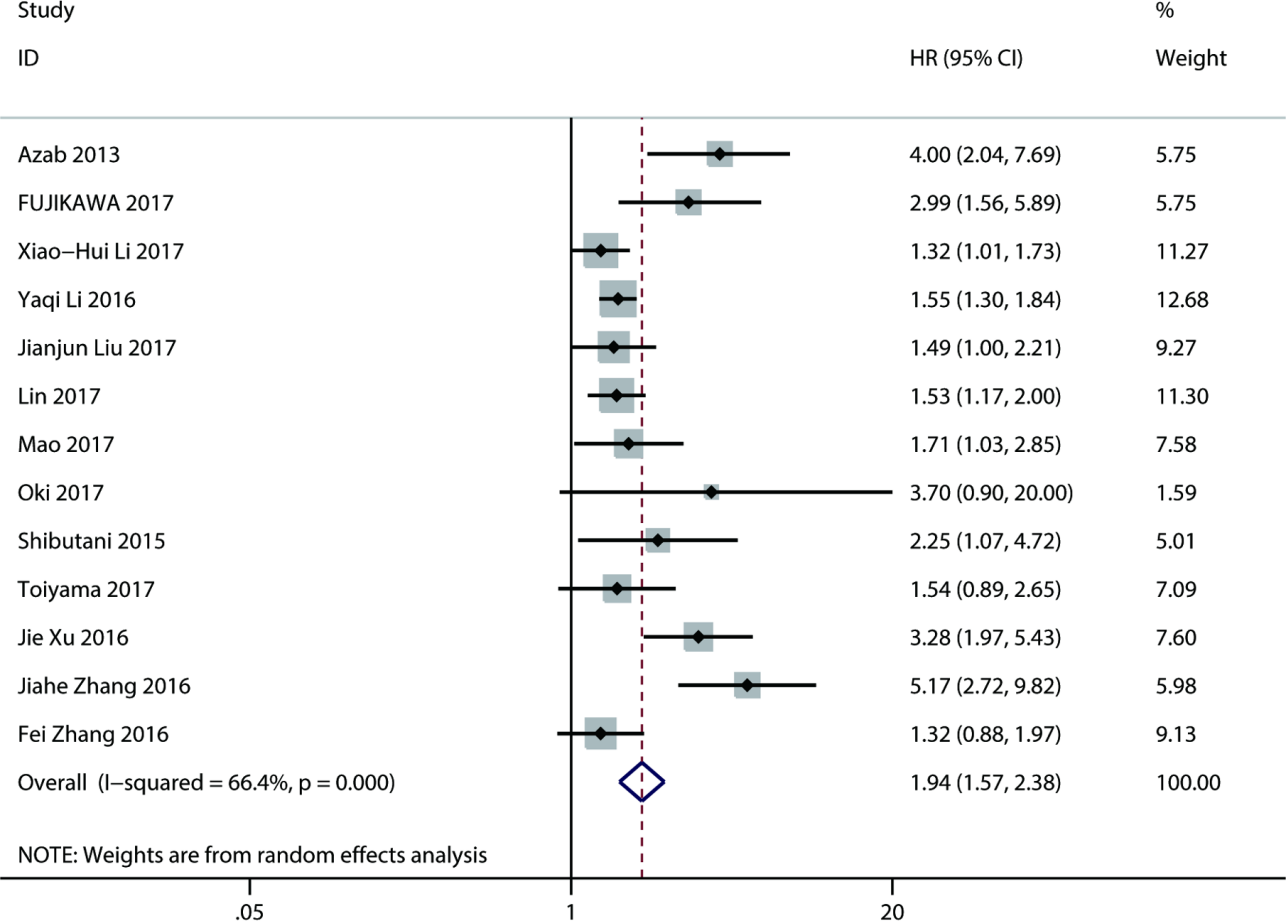
Results of pooled hazard ratios of overall survival of patients with low AGR.

Thirteen studies [8–12, 15–17, 22, 24–27] that included 9269 patients explored the association of the AGR with OS. A random-effect model was used to calculate the HR and 95% CI due to severe heterogeneity (I^2^ = 66.4%, P<0.0001). Pooled analysis showed that a low AGR was significantly connected with poor OS (HR = 1.94; 95% CI: 1.57–2.38; P <0.001) (Fig 2).

Because a substantial heterogeneity existed, subgroup analyses, according to cancer type, region, sample size, cut-off value, cut-off selection, and treatment method, were carried out to explore the potential sources of heterogeneity. As the subgroup analysis results showed (Table 2), a low AGR remained a predictor for worse OS in colorectal cancers (HR = 2.39; 95% CI: 1.45 to 3.94; P = 0.001), esophageal cancers (HR = 1.35; 95% CI: 1.08 to 1.68; P = 0.008), and gastric cancer (HR = 1.56; 95% CI: 1.19 to 2.05; P = 0.001). In addition, we still observed that a low AGR was related to unfavorable OS in China (HR = 1.75; 95% CI: 1.40 to 2.20; P<0.001), Japan (HR = 2.13; 95% CI: 1.49 to 3.04; P<0.001), and the USA (HR = 4.00; 95% CI: 2.06 to 7.77; P<0.001). The correlation of a low AGR with OS was significant according to sample size (≤300 or >300) (HR = 2.76; 95% CI: 1.75 to 4.36; P<0.001 or HR = 1.53; 95% CI: 1.36 to 1.72; P<0.001), cut-off value (≤1.30 or >1.30) (HR = 2.83; 95% CI: 1.80 to 4.45; P<0.001 or HR = 1.51; 95% CI: 1.35 to 1.70; P<0.001), and treatment method (surgery alone or comprehensive treatment) (HR = 1.97; 95% CI: 1.40 to 2.77; P<0.001 or HR = 1.95; 95% CI: 1.45 to 2.61; P<0.001). Furthermore, from the results of the cut-off selection subgroup, we found that a low AGR was significantly associated with unfavorable OS in the ROC group (HR = 1.55; 95% CI: 1.31 to 1.84; P<0.001), X-tile program group (HR = 2.17;95% CI: 1.00 to 4.71; P = 0.049), and for other selection methods (HR = 2.20; 95% CI: 1.25 to 3.28; P = 0.004), but no significance was observed in the cutoff finder group (HR = 2.55; 95% CI: 0.67 to 9.72; P = 0.171).

**Table 2:** Results of subgroup analysis of pooled hazard ratios of overall survival of patients with low AGR.

Stratified analysis	No. of studies	Pooled HR (95% CI)	p-value	Heterogeneity		
				I2 (%)	P-value	Model
[1] Tumor type						
Colorectal cancer	4	2.39(1.45,3.94)	0.001	72.1	0.013	Random effects
Esophageal carcinoma	3	1.35(1.08,1.68)	0.008	0	0.435	Fixed effects
Gastric cancer	3	1.56(1.19,2.05)	0.001	0	0.913	Fixed effects
Cholangiocarcinoma	1	1.53(1.17,2.00)	0.002	-	-	Fixed effects
Pancreatic adenocarcinoma	1	3.28(1.97,5.45)	<0.001	-	-	Fixed effects
Hepatocellular carcinoma	1	5.17(2.72,9.82)	<0.001	-	-	Fixed effects
[2] Country						
China	8	1.75(1.40,2.20)	<0.001	70.6	0.001	Random effects
Japan	4	2.13(1.49,3.04)	<0.001	0	0.411	Fixed effects
USA	1	4.00(2.06,7.77)	<0.001	-	-	Fixed effects
[3] Sample size						
≤ 300	7	2.76(1.75,4.36)	<0.001	71.3	0.004	Random effects
>300	7	1.53(1.36,1.72)	<0.001	39.8	0.126	Fixed effects
[4]Cut-off						
≤1.35	6	2.83(1.80,4.45)	<0.001	72.2	0.003	Random effects
>1.35	7	1.51(1.35,1.70)	<0.001	0	0.860	Fixed effects

**Fig 3:**
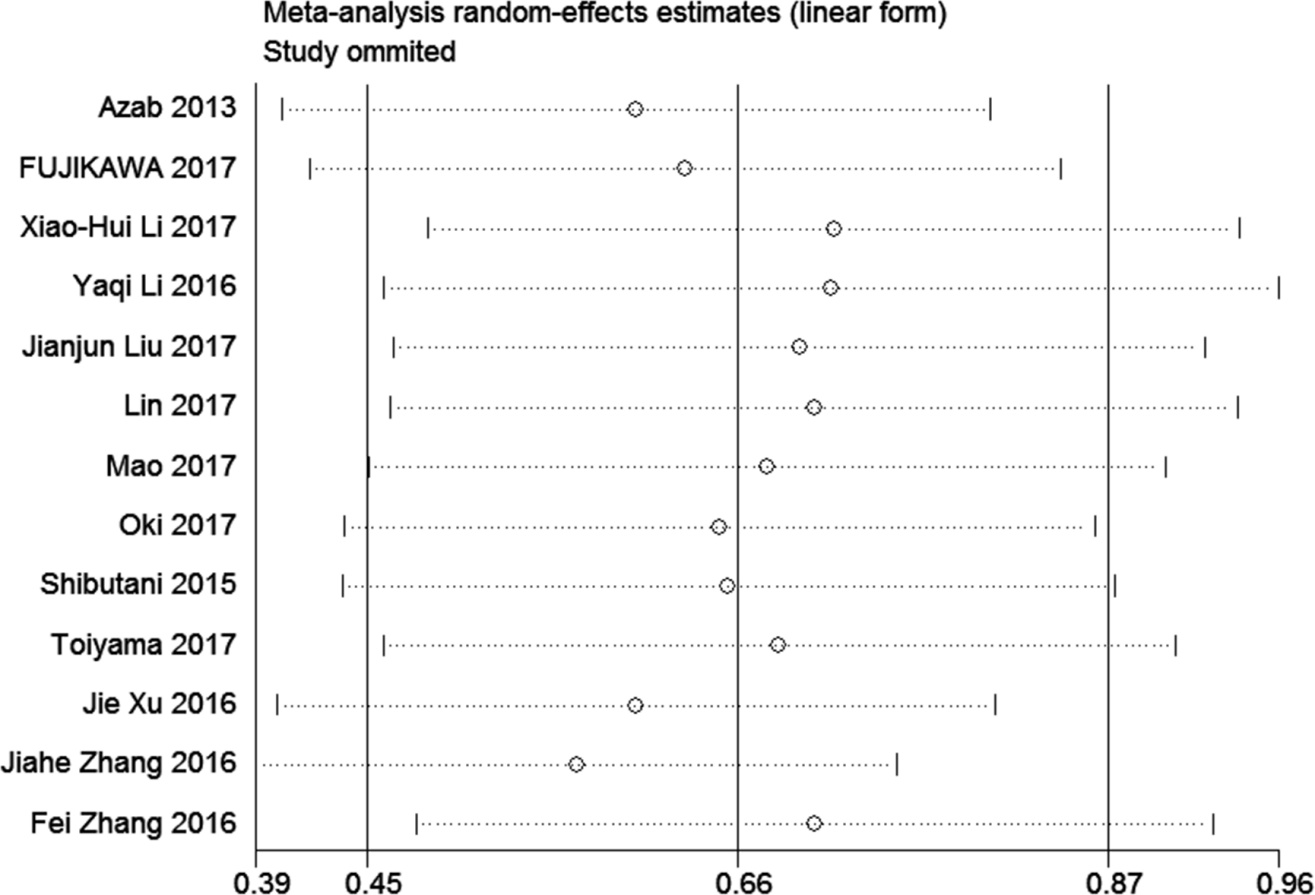
Sensitivity analysis for the pooled hazard ratios of overall survival of patients with low AGR.

**Fig 4:**
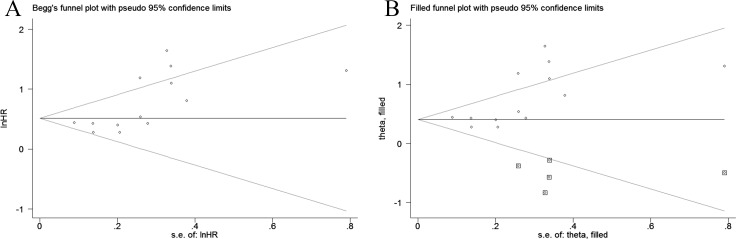
Funnel plots for the evaluation of potential publication bias. (A) Funnel plots depicting the publication bias among the included studies on overall survival. (B) The adjusted funnel plots depicting the publication bias among the included studies on overall survival.

A sensitivity analysis was conducted to evaluate the stability of the pooled HR for OS by omitting one study at each step. The result suggested that the results did not alter substantially (Fig 3), indicating the robustness of the results. Additionally, we assessed the publication bias according to the Begg’s funnel plot and Egger’s test. Nevertheless, the funnel plot was not symmetric (Fig 4A), and a significant publication bias was found by Begg’s test (z = 2.14, P = 0.033) and Egger’s test (t [bias] = 2.91, P = 0.014). Then, the “trim and fill method” was applied to replace five missing studies (Fig 4B), and the adjusted funnel plot was symmetric. Furthermore, after correction, the adjusted pooled HR was 1.521 (95% CI: 1.198–1.929, p = 0.001) based on the random-effect model, which indicated that the publication bias did not significantly influence the reliability of the association of a low AGR with poor OS.

#### A low AGR and disease-free survival (DFS)

**Fig 5:**
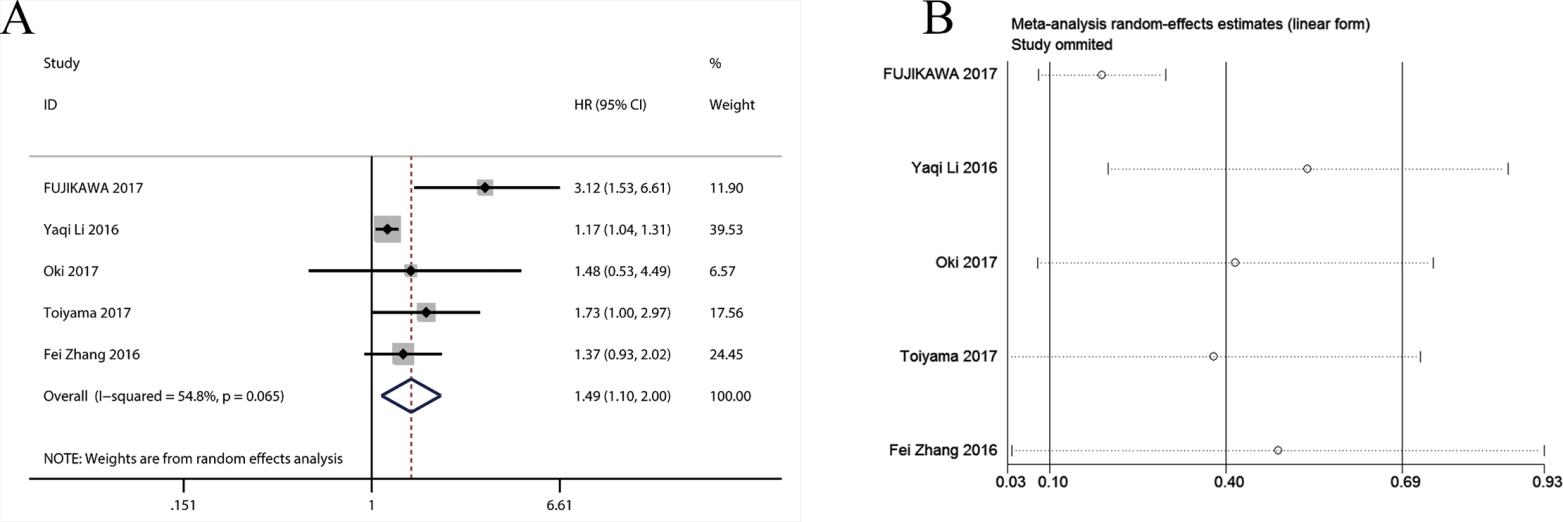
Results of pooled hazard ratios and sensitivity analysis. (A) Results of pooled hazard ratios of diseases-free survival of patients with low AGR. (B) Sensitivity analysis for the pooled hazard ratios of diseases-free survival of patients with low AGR.

The relationship of a low AGR with DFS was reported in 5 studies that included 6538 patients [10, 12, 24, 25, 27]. Because of the obvious heterogeneity, pooled analysis was conducted with a fixed-effect model (I^2^ = 47.2, P = 0.078) and showed that there was a significant association of a low AGR with worse DFS (HR = 1.49; 95% CI: 1.10 to 2.00; P < 0.001) (Fig 5A). In addition, sensitivity analysis was conducted to evaluate the stability of the pooled HR for DFS by excluding a single study in turn. The result showed that the pooled HR was not altered substantially (Fig 5B), suggesting that our result was robust. Publication bias was not applied when analyzing the correlation of a low AGR with DFS due to the limited number of studies that investigated the relationship of a low AGR with DFS.

#### A low AGR and cancer-specific survival (CSS) in digestive system cancers

**Fig 6:**
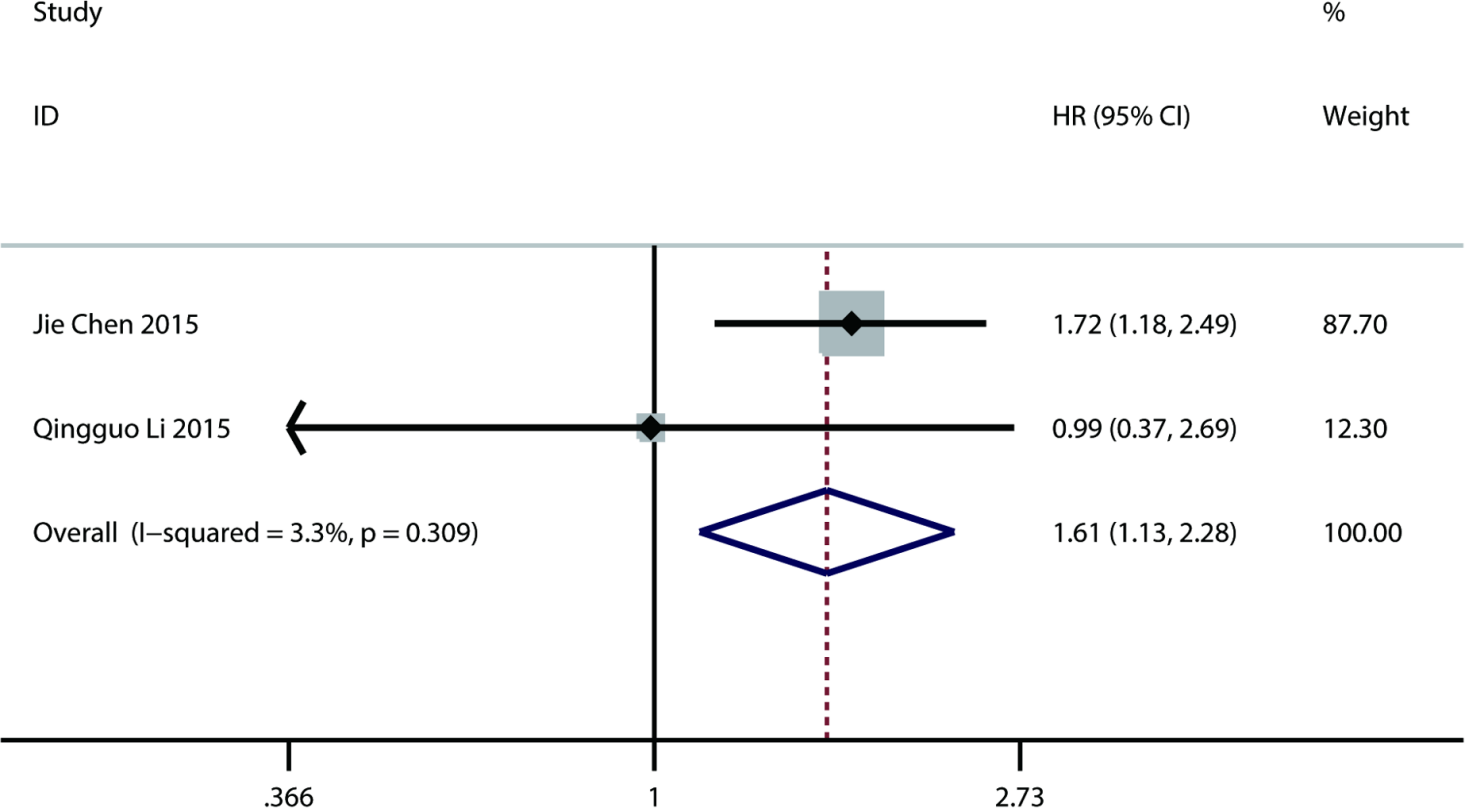
Results of pooled hazard ratios of cancer-specific survival of patients with low AGR.

Only 2 studies enrolling 479 patients explored the association of a low AGR with CSS [23, 28]. A fixed-effect model was applied to calculate the pooled HR with 95% CI due to no significant heterogeneity (I^2^ = 3.3, P = 0.309). The result showed that a low AGR was significantly related to worse CSS (HR = 1.61; 95% CI: 1.13 to 2.28; P = 0.003) (Fig 6). Publication bias and sensitivity analysis were not applicable when analyzing the correlation of a low AGR with CSS due to the limited number of studies investigating the relationship of a low AGR with CSS.

## Discussion

To the best of our knowledge, no meta-analyses have been conducted to date to assess the prognostic value of the AGR in patients with DSCs. In this meta-analysis, data from 15 studies investigating the association of the AGR with the prognosis of patients with DSCs was combined for statistical analysis. The results indicated that a low pretreatment AGR was significantly related to worse survival outcomes in digestive system cancers.

With respect to the potential mechanisms, nutrition and inflammation might be responsible for the prognostic value of the AGR in tumors. In fact, there is a mutual promotion effect between cancer progression and inflammation. Malnutrition commonly occurs in patients with malignancies and slowly leads cancer patients into cachexia, which contributes to disease progression. Cancer-related inflammation refers not only to inflammatory factors produced by malignant cells but also to those generated when injured tissues are remodeled, rehabilitated and vascularized [29]. Furthermore, it has been demonstrated that an abnormal elevation of serum or tumor-local inflammatory cytokines, including tumor necrosis factor; interleukin -1, -6, and -8; and vascular endothelial growth factor, could contribute to cancer progression by promoting tumor growth and metastasis [29, 30]. Thus, effective and practical biomarkers reflecting nutritional and inflammation conditions may be helpful to assess the prognosis of cancer patients. The AGR is generated from the combination of nutritional and inflammatory indices, so it may be a particularly helpful biomarker in this respect. Usually, the serum albumin level is considered to mirror the nutritional condition of the body. Furthermore, recent studies have also demonstrated that the serum albumin level is also able to reflect the body’s inflammatory status [31]. Likewise, the serum globulin level is closely associated with the immune and inflammatory status of the body. It was demonstrated that elevated serum globulin levels caused by the accumulation of acute-phase proteins and immunoglobulins usually reflect a persistent inflammatory response [8, 11]. Serum albumin and globulin levels are easily affected by dehydration and fluid retention, which are relatively common in cancer patients. As such, they fail to reflect the authentic nutritional and inflammatory status of cancer patients. Therefore, the AGR takes the serum albumin and globulin levels into account concurrently, may more precisely mirror the body’s nutritional and inflammatory states and may be particularly helpful to predict the prognosis of cancer patients.

To the best of our knowledge, our study is the first meta-analysis to evaluate the prognostic value of the AGR in patients with digestive system cancer. Although we found that a low pretreatment AGR was closely associated with a worse prognosis of patients with digestive system cancer, several limitations in our meta-analysis should be taken into consideration. First, most of the included studies were designed retrospectively, which unavoidably has a bias risk and thus affects the reliability of our pooled analysis. Second, the cutoff value ranged from 0.9 to 1.93, which may aggravate the heterogeneity among the included studies. Third, the included studies involved in patients’ population from different ethnicity groups, which might also lead to the heterogeneity and limit the generalization of the conclusion from our meta-analysis. Fourth, Egger’s and Begg’s tests indicated that there was a significant publication bias in our meta-analysis, for which the criteria that only published studies were included in our meta-analysis may be partly responsible. This may more or less influence the reliability of our pooled results, although the results of the “trim and fill” method to adjust for the publication bias suggested that the corrected pooled effect size for OS was still statistically significant. Finally, the number of studies included for the pooled estimates of RFS, DFS, CSS and PFS were rather few, and the small sample size may threaten the reliability of the pooled results in that regard. Therefore, the prognostic value of the AGR in patients with digestive system cancer requires further investigation.

In conclusion, our meta-analysis demonstrated that a low pretreatment AGR was closely related to worse long-term outcomes in patients with digestive system cancer. Nevertheless, further large prospective and homogeneous studies should be conducted to validate the prognostic value of the AGR.

## Supporting information

S1 File PRISMA 2009 checklist. (DOC)

S2 File Search criteria. (DOCX)
