# Supplementary material for: Prognostic value of pretreatment albumin/globulin ratio in digestive system cancers: A meta-analysis
Source: PLoS One. 2018 Jan 4;13(1):e0189839. doi: 10.1371/journal.pone.0189839 (PMC5754056; doi:10.1371/journal.pone.0189839)
Supplement: S2 File — (DOCX) [file pone.0189839.s002.docx]

| [#1](https://www.ncbi.nlm.nih.gov/pubmed/advanced) | [Add](https://www.ncbi.nlm.nih.gov/pubmed/advanced) | Search **((((((((((((gastric[Title/Abstract]) OR stomach[Title/Abstract]) OR colon[Title/Abstract]) OR rectal[Title/Abstract]) OR colorectal[Title/Abstract]) OR liver[Title/Abstract]) OR hepatocellular[Title/Abstract]) OR pancreatic[Title/Abstract]) OR esophageal[Title/Abstract]) OR esophagus[Title/Abstract]) OR cholangio*[Title/Abstract]) OR gallbladder[Title/Abstract]) OR bile duct[Title/Abstract]** | 25 |
| --- | --- | --- | --- |
| [#2](https://www.ncbi.nlm.nih.gov/pubmed/advanced) | [Add](https://www.ncbi.nlm.nih.gov/pubmed/advanced) | Search **((((tumor[Title/Abstract]) OR cancer[Title/Abstract]) OR carcinoma[Title/Abstract]) OR adenocarcinoma[Title/Abstract]) OR malignan*[Title/Abstract]** | [2490846](https://www.ncbi.nlm.nih.gov/pubmed/?cmd=HistorySearch&querykey=2) |
| [#3](https://www.ncbi.nlm.nih.gov/pubmed/advanced) | [Add](https://www.ncbi.nlm.nih.gov/pubmed/advanced) | Search **(((albumin to globulin ratio[Title/Abstract]) OR albumin/globulin[Title/Abstract]) OR albumin to globulin[Title/Abstract]) OR AGR[Title/Abstract]** | [2265](https://www.ncbi.nlm.nih.gov/pubmed/?cmd=HistorySearch&querykey=3) |
| [#4](https://www.ncbi.nlm.nih.gov/pubmed/advanced) | [Add](https://www.ncbi.nlm.nih.gov/pubmed/advanced) | Search **((prognosis[Title/Abstract]) OR prognostic[Title/Abstract]) OR survival[Title/Abstract]** | [1099170](https://www.ncbi.nlm.nih.gov/pubmed/?cmd=HistorySearch&querykey=4) |
| [#5](https://www.ncbi.nlm.nih.gov/pubmed/advanced) | [Add](https://www.ncbi.nlm.nih.gov/pubmed/advanced) | Search **((((((((((((((((gastric[Title/Abstract]) OR stomach[Title/Abstract]) OR colon[Title/Abstract]) OR rectal[Title/Abstract]) OR colorectal[Title/Abstract]) OR liver[Title/Abstract]) OR hepatocellular[Title/Abstract]) OR pancreatic[Title/Abstract]) OR esophageal[Title/Abstract]) OR esophagus[Title/Abstract]) OR cholangio*[Title/Abstract]) OR gallbladder[Title/Abstract]) OR bile duct[Title/Abstract])) AND (((((tumor[Title/Abstract]) OR cancer[Title/Abstract]) OR carcinoma[Title/Abstract]) OR adenocarcinoma[Title/Abstract]) OR malignan*[Title/Abstract])) AND ((((albumin to globulin ratio[Title/Abstract]) OR albumin/globulin[Title/Abstract]) OR albumin to globulin[Title/Abstract]) OR AGR[Title/Abstract])) AND (((prognosis[Title/Abstract]) OR prognostic[Title/Abstract]) OR survival[Title/Abstract])** | [27](https://www.ncbi.nlm.nih.gov/pubmed/?cmd=HistorySearch&querykey=5) |
